# A Higher Performance Data Backup Scheme Based on Multi-Factor Authentication †

**DOI:** 10.3390/e26080667

**Published:** 2024-08-05

**Authors:** Lingfeng Wu, Yunhua Wen, Jinghai Yi

**Affiliations:** 1 School of Computer Science and Technology, Donghua University, Shanghai 201620, China; wulingfeng0517@163.com (L.W.); yjh_0206@163.com (J.Y.); 2 State Key Laboratory of Information Security, Institute of Information Engineering, Chinese Academy of Sciences, Beijing 100093, China

**Keywords:** data backup scheme, multi-factor authentication, user-centric deaign, secret sharing scheme

## Abstract

Remote data backup technology avoids the risk of data loss and tampering, and has higher security compared to local data backup solutions. However, the data transmission channel for remote data backup is not secure, and the backup server cannot be fully trusted, so users usually encrypt the data before uploading it to the remote server. As a result, how to protect this encryption key is crucial. We design a User-Centric Design (UCD) data backup scheme based on multi-factor authentication to protect this encryption key. Our scheme utilizes a secret sharing scheme to divide the encryption key into three parts, which are stored in the laptop, the smart card, and the server. The encryption key can be easily reconstructed from any two parts with user’s private information password, identity and biometrics. As long as the biometrics has enough entropy, our scheme can resist replay attacks, impersonation user attacks, impersonation server attacks, malicious servers and offline password guessing attacks.

## 1. Introduction

In recent years, cloud computing technology has developed rapidly, and remote cloud storage services have been widely applied due to their higher security and scalability [1]. However, there is still a risk of data leakage during the data transmission to remote servers [2]. Therefore, people usually encrypt data before transmitting them, such as by using an AES encryption algorithm [3]. In this way, even if the data are leaked, the adversary cannot recover the plaintext. Usually, users store their encryption key in devices such as USBs and laptops, but these devices pose a risk of theft and tampering. Once the storage device is stolen or tampered with, the key will be leaked or lost. How to securely protect the encryption key is the core of remote cloud storage.

By using a (t,n)-threshold secret sharing scheme, people can split the encryption key into *n* shares, and store the *n* shares in different devices. As long as the user obtains *t* shares, the user can reconstruct the encryption key. If the shares are stored in devices in plain text, once *t* devices are corrupt, the encryption key is leaked.

Chang et al. [4] proposed a data protection scheme based on Shamir’s (2,3)-threshold secret sharing scheme to protect sensitive data. In their scheme, the server chooses the encryption key, and divides the key into three shares, which are stored in the laptop, the USB device, and the server, respectively. The user can reconstruct the key on the laptop with the help of the USB offline after the user obtains the authentication of the USB device via their identity and password. If the USB or laptop is unavailable, the user can reconstruct the key through the interaction of the laptop or USB device with the server online after the user obtains the authentication of the server. However, since the encryption key is chosen by the server, it requires the server to be fully trustable. This is infeasible in reality.

To solve the problem in Chang et al’s scheme, Liu et al. [5] proposed a user-centered design (UCD) data backup scheme. In their scheme, the encryption key is chosen by the data owner rather than the server. Similar to Chang et al.’s scheme, using Shamir’s (2,3)-threshold secret sharing scheme, the encryption key is divided into three shares. The user stores the three shares in the laptop, the smart card, and the server, respectively. Whenever the user needs to encrypt/decrypt data, they can reconstruct the key with two of the three devices and their identity, password, and biometrics.

Hu et al. [6] found that the design of Liu et al.’s scheme in the authentication phase is unreasonable, as their scheme can not resist offline password-guessing attacks, server/user camouflage attacks, and so on. Then, Hu et al. presented an enhanced secure data backup scheme to overcome all above-mentioned security threats.

In 2023, Yi et al. [7] found that Hu et al.’s scheme cannot achieve their claimed security. Their scheme could not resist offline guessing attacks, replay attacks, and denial of service attacks. They also did not consider the situation of users rebuilding an incorrect key. Then, Yi et al. proposed an enhanced scheme to address the aforementioned issues. Yi et al. constructed an enhanced data backup scheme based on Shamir’s (2,3)-threshold secret sharing scheme, a message authentication code, and a robust fuzzy extractor. In their scheme, the user chooses the encryption key. Then, by Shamir’s (2,3)-threshold secret sharing scheme, the user divides the encryption key into three shares. The user computes pseudoshares using their private information such as identity, password, and biometrics and stores the three pseudoshares in the laptop, the smart card, and the server, respectively. Whenever the user needs to encrypt/decrypt data, he can reconstruct the key if he gets access to two of the three devices and types the correct private information (identity, password, and biometrics) to pass the multi-factor authentication.

In Yi et al.’s scheme, if the smart card/laptop is unavailable, the user needs to connect their laptop/smart card to the server and provides the valid identity, password, and biometrics to pass the authentication of the server. After the authentication phase, a session key is established between the user and the server. Then, the server uses the session key to encrypt the pseudoshare ($A_{ser}$) stored on the server. Finally, the user obtains two shares and performs the recovery phase to reconstruct the encryption key.

Establishing a session key and using the session key to encrypt the pseudoshare $A_{ser}$ involves more hash computations and more communication rounds between the laptop/smart card and the server.

We are wondering, can we reduce the hash computations and communication rounds between the laptop/smart card and the server?

Our Contribution. We answer the above question in the affirmative. We propose a data backup scheme which inherits the advantages of Yi et al.’s scheme while having higher execution efficiency. More precisely, our contributions are as follows:

- We propose a data backup scheme which fixes two encryption keys $sk_{lps}$ and $sk_{scs}$ shared between the laptop and the server, and the smart card and the server, respectively, during the registration phase. In this way, we can reduce ten hash computations and one round communication in the authentication phase, which improves the execution efficiency of our scheme. We recall that, in Yi et al.’s scheme, there are two communication rounds between the laptop/smart card and the server. This means that we have reduced the number of communication rounds by 50%.
- In addition, our scheme also enjoys other benefits, such as reducing two hash computations and four hash computations during the registration phase and the updating phase, respectively.
- We give the security analysis and performance evaluation of our new data backup scheme, which shows that our new scheme enjoys the same security of previous papers and has a higher execution efficiency.

### 1.1. Related Work

#### 1.1.1. Key Management

Key management is one of the core issues in the field of cryptography, defined as a set of techniques and processes that enable the establishment and maintenance of encrypted key relationships between authorized parties. Under certain security policy controls, it completes the entire process from key generation to final destruction, including key generation, storage, distribution, use, backup, recovery, update, revocation, and destruction. According to their different characteristics, key management can be mainly divided into the following categories.

Dynamic and Static Key Management

Static management adopts the principle of key pre-allocation, which ensures that the keys allocated to each participant throughout the entire lifecycle of the network are fixed. In this way, the key usage time is longer and the probability of being attacked significantly increases. On the contrary, in dynamic key management, encryption keys are updated throughout the entire network lifecycle, which significantly improves the security and lifecycle of the system.

Centralized and Distributed Key Management

Centralized key management refers to a single central node responsible for generating, distributing, and updating encryption keys or session keys used by nodes in the system. Xu et al. proposed a key management scheme based on multi-party joint management [8], which uses an authoritative key generation center and multiple ordinary key generation centers to work together to generate keys. The addition of the authoritative center avoids the problem of malicious nodes randomly modifying selected strings to steal multiple user private keys. The advantage of centralized key management schemes is lower computational and transmission overhead, but it requires a trusted third party to act as the key generation center (KGC) and establish paired shared keys with each user during the registration phase.

In distributed key management, there is no clear key generation center, which can reduce dependence on central entities. Xu et al. [9] proposed a decentralized key management scheme based on a dynamic trust model, which does not require centralized or pre-established trust institutions and introduces three subsystems to integrate dynamic trust and key management. Zheng et al. [10] proposed a decentralized key management scheme based on secret sharing, which divides the key into multiple sub-keys and shares and distributes them to multiple nodes. Multiple nodes jointly maintain one key without a central node. In distributed key management, although the dependence on central entities is reduced, the resources of nodes are limited, and most schemes suffer from overly complex key algorithms and excessive resource consumption.

Symmetric and Asymmetric Key Management

Symmetric key schemes refer to encryption techniques where both parties use the same pair of keys for encryption and decryption, with DES, 3DES, and AES algorithms being the main representatives. Symmetric key schemes have a high speed in encryption and decryption, and can improve the difficulty of information cracking by using long keys. However, the distribution of symmetric keys requires a strictly secure channel, which is difficult to guarantee. Moreover, all nodes that require communication need different key pairs, making the key distribution very difficult in large-scale networks.

Asymmetric key schemes use publicly available public keys and confidential private keys as encryption and decryption key pairs, which have high security and low storage requirements, and better meet the needs of identity authentication in networks. However, asymmetric encryption has lower encryption efficiency and is often combined with symmetric encryption algorithms to form a hybrid encryption scheme that balances security and efficiency.

Many scholars have proposed a series of key negotiation protocols. In 2014, Yang et al. [11] proposed a three-party authentication key protocol for smart cards, which was later proven by Park [12] to be unable to resist offline password-guessing attacks and internal privilege attacks. In 2017, Jiang et al. [13] proposed a three-factor lightweight authentication and key negotiation protocol for wireless sensor networks, but their scheme lacks perfect forward security, resistance to impersonation attacks, and message integrity.

#### 1.1.2. Secure Multi-Party Computation

The basic idea of secure multi-party computation (MPC) was first introduced by Yao in 1982 in the “Millionaire” problem [14]. Afterwards, Goldreich, Micali, and Wigderson [15] extended two-party computation to multi-party computation and provided a security definition for secure multi-party computation. Generally speaking, secure multi-party computation allows a group of untrusted data holders to jointly calculate a predetermined function using their respective secret data as input without relying on any third party. This makes it possible to use secure multi-party computation to build privacy protection applications. The basic cryptographic primitives involved in MPC mainly include Oblivion Transfer (OT), Garbled Circuit (GC), secret sharing (SS), and so on. We mainly introduce the work related to secret sharing.

The secret sharing scheme is a protocol that securely distributes secret information to a certain group of users. Secret sharing can divide secret information into multiple parts and hand them over to different participants, each of whom can only obtain a portion of the information. Only when some participants collaborate together can the complete secret information be recovered. The linear secret sharing scheme (LSSS) refers to a sharing protocol in which a group of users can restore their own shares to their original secret values solely through linear operations. At present, research on secure multi-party computation based on secret sharing mainly focuses on linear secret sharing schemes.

The secret sharing scheme was initially independently proposed by Shamir and Blakley in 1979. The former was constructed based on interpolation polynomials [16], while the latter was based on hyperplane geometry [17]. Subsequently, more threshold secret sharing schemes were proposed, and their properties were continuously improved and strengthened through discussions. The BGW protocol [18] is a classic secure multi-party computation protocol constructed based on the secret sharing protocol. Ben Or and Rabinz [19] proposed the verifiable secret sharing scheme in 1989 and constructed a secure multi-party computation protocol based on it that includes an honest majority of participants.

#### 1.1.3. Multi-Factor Authentication

The existing identity authentication protocols include three basic authentication factors [20]:

- Knowledge factors: what the user knows (such as passwords or PINs);
- Ownership factors: things owned by the user (such as tokens, smart cards, or smartphones);
- Biometric factors: the user’s biometrics (such as fingerprints or iris).

These three basic authentication factors can be used alone or in combination to form an identity authentication system.

Password-based authentication technology began in the 1970s, where the user’s identity and password were stored in the server’s authentication table, which was directly compared with the information on the authentication table when the user logged in. In 1981, Lamport [21] first proposed a password authentication scheme for non-secure channels. Subsequent solutions have been improved in terms of security, computational cost, and effectiveness [22,23,24]. These schemes are easy to implement, but the disadvantage is that the server needs to maintain a password table. Chen Ku [25] pointed out that they are vulnerable to verification table leakage attacks. In 1989, Harn, Huang, and Laih [26] proposed a password authentication scheme based on the public key encryption system. In this scheme, the Diffie Hellman public key [27] encryption technology is used, and the server no longer needs to protect the password table.

With the development of smart cards, people are beginning to attempt to combine smart cards with passwords to address the shortcomings of single-factor authentication schemes. In 1991, Chang and Wu [28] proposed a dual-factor identity authentication protocol based on the Chinese remainder theorem using smart cards. Afterwards, many scholars [29,30,31] conducted extensive research on this dual-factor identity authentication protocol. However, none of these schemes have achieved their claimed security, and dual-factor identity authentication schemes are easily vulnerable to existing internal attacks, anonymity attacks, and other attacks. Particularly, due to the inherent characteristics of smart cards, many schemes are unable to resist the loss of smart cards [32,33].

The emergence of biometric recognition technology has provided a new breakthrough point for identity authentication. Biometrics have many characteristics such as universality, uniqueness, and stability, which provide a more reliable and convenient way of identity verification. In 2002, Lee et al. [34] proposed an identity authentication protocol based on fingerprints and smart cards, which first applied three-factor authentication technology to remote identity authentication protocols. However, biometric technology also has some drawbacks, as users’ biometric information, such as fingerprints, can be easily obtained by adversaries. Furthermore, it is difficult for users to modify their biometric information. Therefore, many protocols [35,36,37] store hashed or encrypted biometric data, rather than the biometric information itself. However, this method is still unrealistic because the recognition data of biometric information are noisy, and the hash function is very sensitive to the input, which can lead to users being unable to successfully complete identity authentication themselves. Fortunately, Dodis et al. [38] proposed the concept of a fuzzy extractor in 2004, which can effectively address this issue.

Nowadays, a multi-factor authentication design for different systems and application scenarios has been widely studied and applied [39,40,41,42,43,44]. For example, Odyuo et al. [39] suggested a novel authentication algorithm based on device serialization and digital signature authentication. According to the suggested approach, a device will only be permitted access to the network if it has successfully completed multi-factor authentication; otherwise, the authentication procedure will fail and must be repeated from scratch. Braeken et al. [40] presented an authentication and key agreement protocol for users who want to have access to constrained sensor nodes deployed in the field, e.g., a doctor with healthcare nodes of a patient. In their protocol, both the sensor and user device provide direct multi-factor authentication relying on physical unclonable functions and biometrics, respectively. In [41], Mostafa et al. proposed an adaptive multi-factor multi-layer authentication framework that embeds an access control and intrusion detection mechanisms with an automated selection of authentication methods. They implemented multiple authentication factors through the user’s geographical location and browser confirmation method that enhance the identity verification of cloud users.

The arrangement of this article is as follows: Section 2 introduces the model of the scheme and some basic tools. In Section 3, we analyze the execution efficiency of Yi et al.’s scheme. In Section 4, we systematically introduce proposed scheme. In Section 5, we conduct a security analysis of the proposed scheme. In addition, we also compare the performance with the scheme proposed by Yi et al. Finally, we provide the conclusion in Section 6.

## 2. Preliminaries

In this chapter, we will first introduce some basic tools used in this paper, and then, we will introduce the model of our scheme. A brief review of Yi et al.’s scheme is given in Appendix A.

Table 1 summarizes the key abbreviations used throughout this paper for ease of reference.

### 2.1. Shamir’s (t-n) Threshold Secret Sharing Scheme

Shamir’s threshold secret sharing scheme is based on the Lagrange interpolation method. It splits the secret *s* and shares it with *n* participants. As long as at least *t* participants cooperate, the secret *s* can be reconstructed. However, as long as there are fewer than *t* participants cooperating, no information about the secret *s* can be obtained. Shamir’s threshold secret sharing scheme includes the following phases:

1. Preparation
  Let GF(p) be a finite field (*p* is a large odd prime number and p>n; *n* is the number of participants), s∈GF(p) is the shared secret, and at least t≤n out of the *n* participants are required to reconstruct *s*.
2. Secret sharing
  Firstly, the secret dealer independently selects t−1 elements $\alpha_{1},\alpha_{2},\ldots ,\alpha_{t-1}\in GF\left(p\right)$, and then constructs a polynomial of degree t−1 as follows:
  (1) $$f\left(x\right)=s+\alpha_{1}x+\alpha_{2}x^{2}+\cdots +\alpha_{t-1}x^{t-1}\left(mod\;p\right),$$
  where Equation (1) satisfies f(0)=s(modp) and $\alpha_{t-1}\neq 0$.
  Then, the dealer randomly selects *n* different non-zero elements $x_{1},x_{2},\ldots ,x_{n}\in GF\left(p\right)$, and computes $y_{i}=f\left(x_{i}\right)$ for i=1,2,…,n. The share $\left(x_{i},y_{i}\right)$ is given to the corresponding participant $P_{i}$.
3. Secret reconstruction
  We assume that the combiner receives *t* shares $\left(x_{i_{1}},y_{i_{1}}\right),\left(x_{i_{2}},y_{i_{2}}\right),\ldots ,\left(x_{i_{t}},y_{i_{t}}\right)$; the polynomial f(x) can be reconstructed by
  (2) $$f\left(x\right)=\sum_{j=1}^{t}y_{i_{j}}\left(\prod_{k=1,k\neq j}^{t}\frac{x-x_{i_{k}}}{x_{i_{j}}-x_{i_{k}}}\right)\left(mod\;p\right),$$
  and then the secret *s* can be recovered, since: s=f(0).

### 2.2. Fuzzy Extractor

A fuzzy extractor can extract a random string *R* from noisy random data with enough entropy, such as biometrics. The extracted random string *R* can be used as the private key or random numbers in the cryptosystem. With the helper string P, it can reproduce the same *R* from biometric templates $Bio^{*}$ and Bio, in case $Bio^{*}$ is sufficiently close to Bio.

**Definition** **1** (Metric spaces)**.** *A metric space is a set W with a distance function $dis:W\times W\to R^{+}=\left[0,\infty \right)$. For all x,y,z∈W, the distance function should satisfy the following conditions:*

*1.*  *Reflexivity: dis(x,y) = 0 if and only if x=y;*
*2.*  *Symmetry: dis(x,y)=dis(y,x);*
*3.*  *Triangle inequality: dis(x,z)≤dis(x,y)+dis(y,z).*

**Definition** **2** (Min-entropy)**.** *For a random variable X, the min-entropy of X, denoted by $H_{\infty }\left(X\right)$, is defined by*

(3) $$H_{\infty }\left(X\right):=-log_{2}\left(max_{x}Pr\left[X=x\right]\right).$$

**Definition** **3** (Robust fuzzy extractor)**.** *A (W,m,ℓ,t,ϵ,δ) robust fuzzy extractor FE consists of two probabilistic polynomial-time algorithms (Gen,Rep). They are described as follows:*

- *(R,P)←Gen(Bio). It takes biometrics Bio∈W as input, and outputs an extracted random string $R\in {\left\{0,1\right\}}^{\ell }$ and an auxiliary string P.*
- *$R\leftarrow Rep(Bio^{*},P)$. The reproduction algorithm Rep takes P and $Bio^{*}\in W$ as inputs, and outputs an extracted string R.*

*Correctness: If $dis(Bio,Bio^{*})\leq t$, then, for all (R,P)←Gen(Bio), it holds that $R\leftarrow Rep(P,Bio^{*})$.*
*Security: Let W be a distribution on W, if $H_{\infty }\left(W\right)\geq m$, then, for all PPT adversaries A,*

(4) $$Adv_{FE,A}^{ind}\left(k\right)=\left|Pr\left[A\left(P,R\right)\Rightarrow 1\right]-Pr\left[A\left(P,U_{\ell }\right)\Rightarrow 1\right]\right|\leq \epsilon ,$$

*where (P,R)←Gen(Bio),Bio←W and $U_{\ell }$ denotes the uniform distribution on ℓ-bit binary strings.*

*Furthermore, these two algorithms satisfy Equation (5) when adversary A involves the following game: A: Compute (R,P)←Gen(Bio) and $\tilde{P}=A\left(R,P\right)$,*

(5) $$Pr(\tilde{P}\neq P\cup Rep\left(Bio^{*},\tilde{P}\right)\neq ⊥)\leq \delta .$$

### 2.3. Message Authentication Code

**Definition** **4** (Message authentication code)**.** *A message authentication code consists of three probabilistic polynomial-time algorithms (Gen,Mac,Vrfy). They are described as follows:*

- *$k\leftarrow Gen(1^{n})$: it takes a security parameter $1^{n}$ as input and outputs a key $k\in {\left\{0,1\right\}}^{n}$.*
- *$t\leftarrow Mac_{k}\left(m\right)$: it takes a key k and a message $m\in {\left\{0,1\right\}}^{*}$ as inputs, and outputs a tag t.*
- *$b\leftarrow Vrfy_{k}\left(m,t\right)$: it takes a key k, a message m, and a tag t as inputs, and outputs a bit b; b=1 means valid and b=0 means invalid.*

*For every n, every $k\in {\left\{0,1\right\}}^{n}$, and every $m\in {\left\{0,1\right\}}^{*}$, it satisfies the following equation:*

(6) $$Vrfy_{k}\left(m,Mac_{k}\left(m\right)\right)=1.$$

**Definition** **5** (Existentially unforgeable under an adaptive chosen-message attack)**.** *A message authentication code Π=(Gen,Mac,Vrfy) is existentially unforgeable under an adaptive chosen-message attack, if, for all probabilistic polynomial-time adversaries A, the message authentication code satisfies the following equation:*

(7) $$Pr[Mac-forge_{A,\Pi }\left(n\right)=1]\leq negl\left(n\right),$$

*where the experiment $Mac-forge_{A,\Pi }\left(n\right)$ is defined as follows:*

1. *A random key $k\leftarrow {\left\{0,1\right\}}^{n}$ is chosen.*
2. *The adversary A is given oracle access to $Mac_{k}\left(.\right)$ and outputs a pair (m,t). Formally, $\left(m,t\right)\leftarrow A^{Mac_{k}\left(.\right)}\left(1^{n}\right)$. Let Q denote the queries asked by A during the execution.*
3. *The output of the experiment is defined to be 1 if and only if $Vrfy_{k}\left(m,t\right)=1$ and m∉Q.*

### 2.4. Model of Our Scheme

Like the scheme proposed by Yi et al., in our model, the user first selects a private key *k* to encrypt the sensitive data, then the user shares the private key *k* among the laptop, the smart card, and the server through Shamir’s (2,3) threshold secret sharing scheme. The private key *k* will be destroyed after the share generation. The user can recover the private key from any two shares using the Lagrange interpolation method. Our scheme mainly focuses on the secret sharing and secret reconstruction phase.

**Figure 1:**
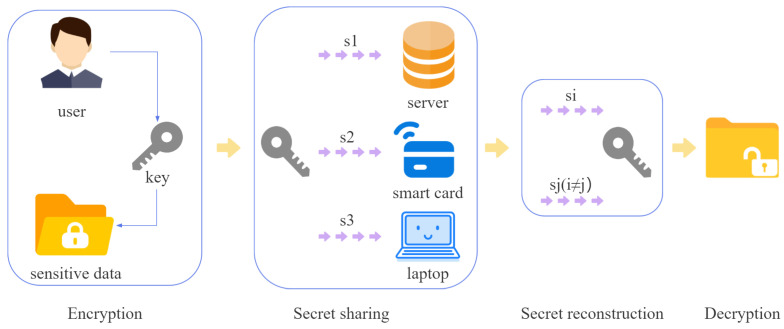
Model of our data backup scheme.

The specific process is shown in Figure 1.

### 2.5. Adversary Capabilities

In this section, we provide a precise description of the adversary’s capabilities.

Capabilities of the adversary

C1: The adversary can eavesdrop, modify, intercept, or redirect the information transferred on open channels.
C2: When the adversary obtains a smart card or a laptop, the adversary can extract the information in it.
C3: The adversary can modify the information on the smart card and the laptop.
C4: The adversary can achieve at most two of the following conditions: (1) obtain the smart card; (2) obtain the laptop; (3) obtain the password; (4) obtain the biometrics; or (5) corrupt the server.

### 2.6. Security Goals

In this section, we describe the security goals of our scheme.

Security goals

T1: Even if the adversary has the above capabilities, the adversary cannot reconstruct the private key *k*.
T2: When the adversary tampers with the smart card or the laptop, the user can quickly detect this attack.
T3: When a malicious server sends a wrong message to the user, the user can quickly detect it.

## 3. Efficiency Analysis of Yi et al.’s Scheme

In this section, we will report the time consumption of each phase in Yi et al.’s scheme. Since the time complexity of the exclusive-OR operation and concatenation operation is negligible, we do not take them into account.

The notations to analyze the computational cost of each phase are as follows:

$T_{H}$: The time complexity of the hash function operation.
$T_{S}$: The time complexity of the symmetric key encryption/decryption operation.
$T_{P}$: The time complexity of the polynomial interpolation operation.
$T_{M}$: The time complexity of the message authentication code tag generation algorithm.
$T_{V}$: The time complexity of the message authentication code verification algorithm.
$T_{G}$: The time complexity of the fuzzy extractor generation algorithm.
$T_{R}$: The time complexity of the fuzzy extractor recovery algorithm.
$T_{SS}$: The time complexity of generating three shares using Shamir’s secret sharing.

Among them, the main notations used to analyze the time complexity of the scheme are $T_{H},T_{S},T_{V}$, and $T_{R}$, while the other notations are only used in the some phases.

The computational cost of three entities at each phase is shown in Table 2. Furthermore, in Table 2, Case 1 is “possession of the laptop and the smart card”, Case 2 is “losing the smart card”, and Case 3 is “losing the laptop”.

## 4. Proposed Scheme

We found that the performance of Yi et al’s scheme is not very ideal. In their scheme, the laptop and the smart card need to establish a session key with the server to obtain the server information, which involves a lot of hash operations. In addition, each updating phase requires going through the above steps.

Therefore, we proposed a more efficient solution: during the registration phase, the server negotiates an encryption key with the laptop and the smart card, respectively. In this way, we can reduce the computation required to establish a session key during the authentication phase, and improve the execution efficiency of the scheme.

In this chapter, we will introduce our more efficient data backup scheme, which consists of four phases: the registration phase, the authentication phase, the key reconstruction phase, and the updating phase.

Similar to Yi et al.’s scheme, in our scheme, the registration phase and key reconstruction phase are carried out on a secure channel. The interaction between the laptop and the smart cards occurs over a secure physical channel. Additionally, all other phases are carried out on the common channel.

The adversary capabilities and the security goals are consistent with those introduced in Section 2; therefore, they are omitted here.

### 4.1. Registration Phase

In the registration phase, the user uses the laptop to interact with the smart card and server to complete the distribution and storage of keys. The specific process is as follows (see Game 1):
**Game 1** Registration phaseSmart card
Laptop (user)
Server

choose

      $ID_{usr},ID_{sc},ID_{ser},$


      Pwd,Bio,ku,


      $f\left(x\right)=rx+k,sk_{lps},sk_{scs};$


compute

      $ID_{usr},ID_{sc},ID_{ser},$


      (R,P)←Gen(Bio),


        R=(R1,R2),


      $y_{usr}=f\left(ID_{usr}\right)$,


      $y_{sc}=f\left(ID_{sc}\right)$,


      $y_{ser}=f\left(ID_{ser}\right)$,


      $A_{usr}=y_{usr}⊕Pwd⊕R1$,


      $A_{sc}=y_{sc}⊕Pwd⊕R1$,


      $A_{ser}=y_{ser}⊕Pwd⊕R1$,


        $V=h(ID_{sc}||Pwd||ID_{usr}||R1||ku)$,


        $H=h(ID_{usr}||Pwd||R1)$,


      $t_{usr}\leftarrow Mac_{R2}\left(A_{usr}\right),$


      $t_{sc}\leftarrow Mac_{R2}\left(A_{sc}\right),$


      $t_{ser}\leftarrow Mac_{R2}\left(A_{ser}\right);$



$\overset{\left(ID_{ser},ID_{sc},ID_{usr},A_{ser}\right)}{\underset{\left(t_{ser},sk_{lps},sk_{scs},H\right)}{\to }}$



choose    X;



compute    $Y=h(ID_{ser}||X),$



               Z=Y⊕H;



store



      $\left(ID_{ser},ID_{sc},ID_{usr}\right)$



and $\left(A_{ser},t_{ser},sk_{lps},sk_{scs},X\right)$


$\overset{Z}{\underset{\;}{\leftarrow }}\;$


store



      $\left(ID_{usr},A_{usr},t_{usr}\right)$



and $\left(sk_{lps},Z,P,ku\right)$


$\overset{\left(ID_{sc},A_{sc},t_{sc}\right)}{\underset{\left(sk_{scs},Z,P,V\right)}{\leftarrow }}$


store



      $\left(ID_{sc},A_{sc},t_{sc}\right)$



and $\left(sk_{scs},Z,P,V\right)$

1. The user chooses $ID_{usr},ID_{sc},ID_{ser},Pwd,Bio$, a random number ku, and a first-degree polynomial f(x)=rx+k, where *k* is the key to encrypt sensitive information, and *r* is a random number. The user chooses a symmetric encryption key $sk_{lps}$ for encrypted communication between the laptop and the server. The user chooses a symmetric encryption key $sk_{scs}$ for encrypted communication between the smart card and the server (e.g.,the user can choose $sk_{lps}$ and $sk_{scs}$ by using the key generation algorithm in AES).
2. The user computes (R,P)←Gen(Bio), and divides *R* into two parts R1,R2, which are, respectively, applied to the three pseudoshares and the message authentication code. The user computes three true shares $y_{usr}=f\left(ID_{usr}\right)$, $y_{sc}=f\left(ID_{sc}\right)$, and $y_{ser}=f\left(ID_{ser}\right)$, three pseudoshares $A_{usr}=y_{usr}⊕Pwd⊕R1$, $A_{sc}=y_{sc}⊕Pwd⊕R1$, and $A_{ser}=y_{ser}⊕Pwd⊕R1$, the authentication message $V=h(ID_{sc}||Pwd||ID_{usr}||R1||ku)$, $H=h(ID_{usr}||Pwd||R1)$, and three tags $t_{usr}\leftarrow Mac_{R2}\left(A_{usr}\right)$, $t_{sc}\leftarrow Mac_{R2}\left(A_{sc}\right)$, and $t_{ser}\leftarrow Mac_{R2}\left(A_{ser}\right)$.
3. The user sends the message $\left(ID_{ser},ID_{sc},ID_{usr},A_{ser},t_{ser},sk_{lps},sk_{scs},H\right)$ to the server.
4. After the server receives the message $\left(ID_{ser},ID_{sc},ID_{usr},A_{ser},t_{ser},sk_{lps},sk_{scs},H\right)$, the server chooses a random number *X* and generates $Y=h(ID_{ser}||X)$, Z=Y⊕H. Finally, the server stores values $\left(ID_{ser},ID_{sc},ID_{usr},A_{ser},t_{ser},sk_{lps},sk_{scs},X\right)$.
5. The server sends the message *Z* to the user.
6. The user stores values $\left(ID_{usr},A_{usr},t_{usr},sk_{lps},Z,P,ku\right)$ in the laptop.
7. The user then sends $\left(ID_{sc},A_{sc},t_{sc},sk_{scs},Z,P,V\right)$ to the smart card.
8. The smart card stores $\left(ID_{sc},A_{sc},t_{sc},sk_{scs},Z,P,V\right)$.

### 4.2. Authentication Phase

If the user wants to recover the private key for decrypting the encrypted sensitive data, the user needs to first perform the authentication phase. According to the possession of secret shares, the authentication phase can be divided into the following threes case: (1) laptop and smart card; (2) laptop and server; and (3) smart card and server.

#### 4.2.1. Laptop and Smart Card

The user can easily obtain the information needed to reconstruct the private key if he has the smart card and the laptop. The specific process is as follows (see Game 2):
**Game 2** Authentication phase: possession of the laptop and the smart cardSmart card
Laptop (user)

compute

      $R^{*}\leftarrow Rep\left(Bio^{*},P\right),$

      $R^{*}=\left(R1^{*},R2^{*}\right),$

      $b\leftarrow Vrfy_{R2^{*}}\left(t_{usr},A_{usr}\right);$

if    b=0, stop;
$\overset{\;(ID_{usr},Pwd,ku,R1^{*},R2^{*})\;}{\underset{\;\;\;\;\;\;\;}{\leftarrow }}$
compute

      $b\leftarrow Vrfy_{R2^{*}}\left(t_{sc},A_{sc}\right);$

if    b=0, stop;

else compute

      $V^{*}=h(ID_{sc}||Pwd||ID_{usr}||R1^{*}||ku);$

check    $V^{*}?=V$

if    $V^{*}\neq V$, stop;


$\overset{A_{sc}}{\underset{\;}{\to }}$


compute

      $y_{sc}=A_{sc}⊕Pwd⊕R1^{*},$

      $y_{usr}=A_{usr}⊕Pwd⊕R1^{*};$

1. The user inserts the smart card into the card reader, and the card reader reads the information $(ID_{sc},A_{sc},t_{sc},sk_{scs},Z,P,V$) in the smart card.
2. The user inputs $Bio^{*}$, and computes $Rep\left(Bio^{*},P\right)=R^{*}$, then divides $R^{*}$ into two parts $R1^{*},$ and $R2^{*}$. Then, the laptop verifies whether $t_{usr}$ is a valid tag for message $A_{usr}$ ($b\leftarrow Vrfy_{R2^{*}}\left(t_{usr},A_{usr}\right)$). If b=0, the laptop stops; otherwise, the laptop sends message $\left(ID_{usr},Pwd,ku,R1^{*},R2^{*}\right)$ to the smart card.
3. After receiving the message, the smart card verifies whether $t_{sc}$ is a valid tag for message $A_{sc}$ ($b\leftarrow Vrfy_{R2^{*}}\left(t_{sc},A_{sc}\right)$). If b=0, the smart card stops.
4. The smart card computes $V^{*}=h(ID_{sc}||Pwd||ID_{usr}||R1^{*}\left||ku\right)$, then checks if $V^{*}$ is equal to *V*.
5. If $V^{*}\neq V$, the procedure aborts; otherwise, the smart card sends $A_{sc}$ to the laptop.
6. After receiving $A_{sc}$, the user computes $y_{sc}^{*}=A_{sc}⊕Pwd⊕R1^{*}$, and $y_{usr}^{*}=A_{usr}⊕Pwd⊕R1^{*}$.

#### 4.2.2. Laptop and Server

When the user’s smart card share is unavailable (e.g., the smart card may be lost, the share may be corrupt, etc.), the user needs to complete the authentication phase through the interaction between the laptop and the server. The specific process is as follows (see Game 3):
**Game 3** Authentication phase: laptop and serverLaptop (user)
Serverinput    $ID_{usr},Pwd,Bio^{*},T1;$

compute      $R^{*}\leftarrow Rep\left(Bio^{*},P\right),$

      $R^{*}=\left(R1^{*},R2^{*}\right),$

      $H=h(ID_{usr}||Pwd||R1^{*}),$

      Y=Z⊕H,

      $c1\leftarrow E_{sk_{lps}}\left(Y\right);$


$\overset{\;(c1,ID_{usr},T1)\;}{\underset{\;}{\to }}$check    $T^{*}-T1?\leq \Delta t$

if    $T^{*}-T1>\Delta t$, stop;

compute

      $Y^{*}\leftarrow D_{sk_{lps}}\left(c1\right),$

      $Y=h(ID_{ser}||X);$

check    $Y^{*}?=Y$

if    $Y^{*}\neq Y$, stop;

otherwise, server authenticates user;

compute

      $c2\leftarrow E_{sk_{lps}}\left(A_{ser}\right);$
$\overset{\;(c2,t_{ser})\;}{\underset{\;}{\leftarrow }}$
compute

      $A_{ser}^{\prime }\leftarrow D_{sk_{lps}}\left(c2\right),$

      $b\leftarrow Vrfy_{R2^{*}}\left(A_{ser}^{\prime },t_{ser}\right);$

if    b=0, stop;

else, compute

      $y_{ser}=A_{ser}^{\prime }⊕R1^{*}⊕Pwd,$

      $y_{usr}=A_{usr}^{\prime }⊕R1^{*}⊕Pwd;$

1. The user inputs $Bio^{*}$, computes $Rep\left(Bio^{*},P\right)=R^{*}$, and divides $R^{*}$ into two parts $R1^{*}$ and $R2^{*}$. Then, the user inputs their $ID_{usr}$, Pwd, and current timestamp T1. The laptop computes $H=h(ID_{usr}||Pwd||R1^{*})$, and Y=Z⊕H, $c1\leftarrow E_{sk_{lps}}\left(Y\right)$.
2. The laptop sends $\left(c1,ID_{usr},T1\right)$ to the server.
3. After receiving the request message $\left(c1,ID_{usr},T1\right)$, the server checks whether the current timestamp $T1^{*}-T1\leq \Delta t$ or not. If $T1^{*}-T1>\Delta t$, the server stops; otherwise, the server uses the symmetric key $sk_{lps}$ to compute $Y^{*}\leftarrow D_{sk_{lps}}\left(c1\right)$.
4. $Y=h(ID_{ser}||X)$. The server check whether $Y^{*}$ is equal to *Y*. If $Y^{*}\neq Y$, the server stops; otherwise, the server authenticates the user identity.
5. The server computes $c2\leftarrow E_{sk_{lps}}\left(A_{ser}\right)$ and sends $\left(c2,t_{ser}\right)$ to the laptop.
6. After receiving the message $\left(c2,t_{ser}\right)$, the laptop computes $A_{ser}^{\prime }\leftarrow D_{sk_{lps}}\left(c2\right)$ and $b\leftarrow Vrfy_{R2^{*}}\left(A_{ser}^{\prime },t_{ser}\right)$. If b=0, the laptop stops; otherwise, the user computes $y_{ser}=A_{ser}^{\prime }⊕R1^{*}⊕Pwd$.

#### 4.2.3. Smart Card and Server

When the user’s laptop share is unavailable (e.g., the laptop may be lost, the share may be corrupt, etc.), the user can use another device with a smart card reader to interact with the server and complete the authentication phase. The specific process is as follows (see Game 4):
**Game 4** Authentication phase: smart card and serverSmart card (user)
Serverinput    $ID_{usr},Pwd,Bio^{*},T1;$

compute

      $R^{*}\leftarrow Rep\left(Bio^{*},P\right),$

      $R^{*}=\left(R1^{*},R2^{*}\right),$

      $H=h(ID_{usr}||Pwd||R1^{*}),$

      Y=Z⊕H,

      $c1\leftarrow E_{sk_{scs}}\left(Y\right);$


$\overset{\;(c1,ID_{sc},T1)\;}{\underset{\;}{\to }}$


check    $T^{*}-T1?\leq \Delta t$

if    $T^{*}-T1>\Delta t$, stop;

compute

      $Y^{*}\leftarrow D_{sk_{scs}}\left(c1\right),$

      $Y=h(ID_{ser}||X);$

check    $Y^{*}?=Y$

if    $Y^{*}\neq Y$, stop;

otherwise, server authenticates user;

compute

      $c2\leftarrow E_{sk_{scs}}\left(A_{ser}\right);$
$\overset{\;\;(c2,t_{ser})\;\;}{\underset{\;}{\leftarrow }}$
compute

      $A_{ser}^{\prime }\leftarrow D_{sk_{scs}}\left(c2\right),$

      $b\leftarrow Vrfy_{R2^{*}}\left(A_{ser}^{\prime },t_{ser}\right);$

if    b=0, stop;

else, compute

      $y_{ser}=A_{ser}^{\prime }⊕R1^{*}⊕Pwd,$

      $y_{usr}=A_{usr}^{\prime }⊕R1^{*}⊕Pwd;$

1. The user inputs $Bio^{*}$, computes $Rep\left(Bio^{*},P\right)=R^{*}$, and divides $R^{*}$ into two parts $R1^{*}$ and $R2^{*}$. Then, the user inputs their $ID_{usr}$, Pwd, and current timestamp T1. The laptop computes $H=h(ID_{usr}||Pwd||R1^{*})$ and Y=Z⊕H, $c1\leftarrow E_{sk_{lps}}\left(Y\right)$.
2. The laptop sends $\left(c1,ID_{sc},T1\right)$ to the server.
3. After receiving the request message $\left(c1,ID_{sc},T1\right)$, the server checks whether the current timestamp $T1^{*}-T1\leq \Delta t$ or not. If $T1^{*}-T1>\Delta t$, the server stops; otherwise, the server uses the symmetric key $sk_{scs}$ to compute $Y^{*}\leftarrow D_{sk_{scs}}\left(c1\right)$.
4. $Y=h(ID_{ser}||X)$. The server check whether $Y^{*}$ is equal to *Y*. If $Y^{*}\neq Y$, the server stops; otherwise, the server authenticates the user identity.
5. The server computes $c2\leftarrow E_{sk_{scs}}\left(A_{ser}\right)$ and sends $\left(c2,t_{ser}\right)$ to the laptop.
6. After receiving the message $\left(c2,t_{ser}\right)$, the laptop computes $A_{ser}^{\prime }\leftarrow D_{sk_{scs}}\left(c2\right)$ and $b\leftarrow Vrfy_{R2^{*}}\left(A_{ser}^{\prime },t_{ser}\right)$. If b=0, the laptop stops; otherwise, the user computes $y_{ser}=A_{ser}^{\prime }⊕R1^{*}⊕Pwd$.

### 4.3. Key Reconstruction Phase

The user can easily recover key *k* through the Lagrange interpolation method if he knows two of the three tuples $\left(ID_{usr},y_{usr}\right),\left(ID_{sc},y_{sc}\right)$, and $\left(ID_{ser},y_{ser}\right)$. For example, if the user knows $\left(ID_{sc},y_{sc}\right)$ and $\left(ID_{usr},y_{usr}\right)$, the user can obtain the private key by computing $k=y_{sc}\left(-ID_{sc}/\left(ID_{usr}-ID_{sc}\right)\right)+y_{usr}\left(-ID_{usr}/\left(ID_{sc}-ID_{usr}\right)\right)\left(mod\;p\right)$.

### 4.4. Updating Phase

When the user has both the smart card and the laptop, the user can update their password or biometrics. When the user’s smart card share or laptop share is unavailable, the user can rebuild it by interacting with the server through another share.

#### 4.4.1. Updating Password

The user can update the password Pwd to $Pwd^{*}$. Before updating the password, the user need to interact with the server through the laptop or the smart card to obtain $A_{ser}$ stored on the server (Section 4.4.2, lines 5 and 6; Section 4.4.3, lines 5 and 6). After obtaining the information $A_{ser}$ stored on the server, the subsequent process is as follows (see Game 5):
**Game 5** Updating passwordSmart card
Laptop (user)
Server

compute



      R←Rep(Bio,P),R=(R1,R2);


$\overset{\left(ID_{usr},Pwd,ku,R1\right)}{\underset{\;}{\leftarrow }}$


compute



      $V^{*}=$



$h(ID_{sc}||Pwd||ID_{usr}||R1||ku);$



check    $V^{*}?=V$



if    $V^{*}\neq V$, stop;




$\overset{\;A_{sc}\;}{\underset{\;}{\to }}$




choose    $ku^{*},Pwd^{*};$



compute



       $V^{*}=h(ID_{sc}||Pwd^{*}||ID_{usr}||R1||ku^{*})$,



       $H^{*}=h(ID_{usr}||Pwd^{*}\left||R1\right)$,



        $H=h(ID_{usr}||Pwd||R1)$,



       $Z^{*}=Z⊕H⊕H^{*}$,



     $A_{usr}^{*}=A_{usr}⊕Pwd⊕Pwd^{*}$,



       $A_{sc}^{*}=A_{sc}⊕Pwd⊕Pwd^{*}$,



      $A_{ser}^{*}=A_{ser}⊕Pwd⊕Pwd^{*}$,



$t_{usr}^{*}\leftarrow Mac_{R2}\left(A_{usr}^{*}\right),t_{sc}^{*}\leftarrow Mac_{R2}\left(A_{sc}^{*}\right),$



$t_{ser}^{*}\leftarrow Mac_{R2}\left(A_{ser}^{*}\right),c\leftarrow E_{sk_{lps}}\left(A_{ser}^{*},A_{ser}\right);$



store



      $\left(Z^{*},ku^{*},A_{usr}^{*},t_{usr}^{*}\right)$


$\overset{\left(Z^{*},V^{*},A_{sc}^{*},t_{sc}^{*}\right)}{\underset{\;}{\leftarrow }}$


store



      $\left(Z^{*},V^{*},A_{sc}^{*},t_{sc}^{*}\right)$






$\overset{\;(t_{ser}^{*},c)\;}{\underset{\;}{\to }}$




compute



      $D_{sk_{lps}}\left(c\right)=\left(A_{ser}^{*},A_{ser}^{\prime }\right);$



if    $A_{ser}^{\prime }\neq A_{ser}$, stop;



store



      $\left(t_{ser}^{*},A_{ser}^{*}\right)$

1. The user inserts the smart card into the card reader, and the card reader reads the information in the smart card.
2. The user obtains $ID_{usr}$ and the random number ku in the laptop, and inputs their biometrics Bio and the old password Pwd. Then, the user computes (R,P)←Gen(Bio) and divides *R* into two parts R1 and R2. After that, the laptop sends $\left(ID_{usr},Pwd,ku,R1\right)$ to the smart card.
3. After receiving the message $\left(ID_{usr},Pwd,ku,R1\right)$, the smart card computes $V^{*}=h(ID_{sc}||Pwd||ID_{usr}\left||R1||ku\right)$ and checks whether $V^{*}$ is equal to *V*. If $V^{*}\neq V$, the smart card stops; otherwise, the smart card sends $A_{sc}$ to the laptop.
4. The user chooses a new random number $ku^{*}$ and a new password $Pwd^{*}$ and computes following values:
  $V^{*}=h(ID_{sc}||Pwd^{*}||ID_{usr}||R1||ku^{*})$, $H^{*}=h(ID_{usr}||Pwd^{*}\left||R1\right)$,
  $H=h(ID_{usr}||Pwd||R1)$, $Z^{*}=Z⊕H⊕H^{*}$,
  $A_{usr}^{*}=A_{usr}⊕Pwd⊕Pwd^{*}$, $A_{sc}^{*}=A_{sc}⊕Pwd⊕Pwd^{*}$, $A_{ser}^{*}=A_{ser}⊕Pwd⊕Pwd^{*}$,
  $t_{usr}^{*}\leftarrow Mac_{R2}\left(A_{usr}^{*}\right)$, $t_{sc}^{*}\leftarrow Mac_{R2}\left(A_{sc}^{*}\right)$, $t_{ser}^{*}\leftarrow Mac_{R2}\left(A_{ser}^{*}\right)$.
  Then, the user stores $\left(Z^{*},ku^{*},A_{usr}^{*},t_{usr}^{*}\right)$ in the laptop to replace $\left(Z,ku,A_{usr},t_{usr}\right)$ and sends $\left(Z^{*},V^{*},A_{sc}^{*},t_{sc}^{*}\right)$ to the smart card.
5. The smart card stores $\left(Z^{*},V^{*},A_{sc}^{*},t_{sc}^{*}\right)$ to replace $\left(Z,V,A_{sc},t_{sc}\right)$.
6. The laptop sends $\left(t_{ser}^{*},c=E_{sk_{lps}}\left(A_{ser}^{*},A_{ser}\right)\right)$ to the server.
7. After receiving the message $\left(t_{ser}^{*},c\right)$, the server calculates $D_{sk_{lps}}\left(c\right)=\left(A_{ser}^{*},A_{ser}^{\prime }\right)$ and checks if $A_{ser}^{\prime }$ is equal to $A_{ser}$. If $A_{ser}^{\prime }=A_{ser}$, the server replaces $\left(A_{ser},t_{ser}\right)$ with $\left(A_{ser}^{*},t_{ser}^{*}\right)$; otherwise, the server stops.

#### 4.4.2. Updating Biometrics

The process of changing the biometrics is similar to that of changing the password. Before updating the biometrics, the user needs to interact with the server through the laptop or the smart card to obtain $A_{ser}$ stored on the server (Section 4.4.2, lines 5 and 6; Section 4.4.3, lines 5 and 6). Furthermore, the subsequent process is as follows (see Game 6):

1. The user inserts the smart card into the card reader, and the card reader reads the information in the smart card.
2. The user obtains $ID_{usr}$ and the random number ku in the laptop, and inputs their biometrics Bio and the password Pwd. Then, the user computes (R,P)←Gen(Bio) and divides *R* into two parts R1 and R2. After that, the laptop sends $\left(ID_{usr},Pwd,ku,R1\right)$ to the smart card.
3. After receiving the message $\left(ID_{usr},Pwd,ku,R1\right)$, the smart card computes $V^{*}=h(ID_{sc}||Pwd||ID_{usr}\left||R1||ku\right)$ and checks whether $V^{*}$ is equal to *V*. If $V^{*}\neq V$, the smart card stops; otherwise, the smart card sends $A_{sc}$ to the laptop.
4. The user chooses a new random number $ku^{*}$ and inputs new biometrics $Bio^{*}$ and computes following values:
  $\left(R^{*},P^{*}\right)\leftarrow Gen\left(Bio^{*}\right)$, $R^{*}=\left(R1^{*},R2^{*}\right)$,
  $V^{*}=h(ID_{sc}||Pwd||ID_{usr}||R1^{*}||ku^{*})$,
  $H^{*}=h(ID_{usr}||Pwd||R1^{*})$, $H=h(ID_{usr}||Pwd||R1)$, $Z^{*}=Z⊕H⊕H^{*}$,
  $A_{usr}^{*}=A_{usr}⊕R1⊕R1^{*}$, $A_{sc}^{*}=A_{sc}⊕R1⊕R1^{*}$, $A_{ser}^{*}=A_{ser}⊕R1⊕R1^{*}$,
  $t_{usr}^{*}\leftarrow Mac_{R2^{*}}\left(A_{usr}^{*}\right)$, $t_{sc}^{*}\leftarrow Mac_{R2^{*}}\left(A_{sc}^{*}\right)$, $t_{ser}^{*}\leftarrow Mac_{R2^{*}}\left(A_{ser}^{*}\right)$.
  Then, the user stores $\left(Z^{*},ku^{*},P^{*},A_{usr}^{*},t_{usr}^{*}\right)$ in the laptop to replace $\left(Z,ku,P,A_{usr},t_{usr}\right)$ and sends $\left(Z^{*},V^{*},P^{*},A_{sc}^{*},t_{sc}^{*}\right)$ to the smart card.
5. The smart card stores $\left(Z^{*},V^{*},P^{*},A_{sc}^{*},t_{sc}^{*}\right)$ to replace $\left(Z,V,P,A_{sc},t_{sc}\right)$.
6. The laptop sends $\left(t_{ser}^{*},c=E_{sk_{lps}}\left(A_{ser}^{*},A_{ser}\right)\right)$ to the server.
7. After receiving the message $\left(t_{ser}^{*},c\right)$, the server calculates $D_{sk_{lps}}\left(c\right)=\left(A_{ser}^{*},A_{ser}^{\prime }\right)$ and checks if $A_{ser}^{\prime }$ is equal to $A_{ser}$. If $A_{ser}^{\prime }=A_{ser}$, the server replaces $\left(A_{ser},t_{ser}\right)$ with $\left(A_{ser}^{*},t_{ser}^{*}\right)$; otherwise, the server stops.

**Game 6** Updating biometricsSmart card
Laptop (user)
Server

compute



      R←Rep(Bio,P),R=(R1,R2);


$\overset{\;(ID_{usr},Pwd,ku,R1)\;}{\underset{\;}{\leftarrow }}$


compute



      $V^{*}=$



$h(ID_{sc}||Pwd||ID_{usr}||R1||ku);$



check    $V^{*}?=V$



if    $V^{*}\neq V$, stop;




$\overset{\;\;A_{sc}\;\;}{\underset{\;}{\to }}$




choose    $ku^{*},Bio^{*};$



compute



      $\left(R^{*},P^{*}\right)\leftarrow Gen\left(Bio^{*}\right),$



       $R^{*}=\left(R1^{*},R2^{*}\right)$,



       $V^{*}=h(ID_{sc}||Pwd||ID_{usr}||R1^{*}||ku^{*})$,



       $H^{*}=h(ID_{usr}||Pwd||R1^{*})$,



        $H=h(ID_{usr}||Pwd||R1)$,



       $Z^{*}=Z⊕H⊕H^{*}$,



     $A_{usr}^{*}=A_{usr}⊕R1⊕R1^{*}$,



      $A_{sc}^{*}=A_{sc}⊕R1⊕R1^{*}$,



      $A_{ser}^{*}=A_{ser}⊕R1⊕R1^{*}$,



$t_{usr}^{*}\leftarrow Mac_{R2^{*}}\left(A_{usr}^{*}\right),t_{sc}^{*}\leftarrow Mac_{R2^{*}}\left(A_{sc}^{*}\right),$



$t_{ser}^{*}\leftarrow Mac_{R2^{*}}\left(A_{ser}^{*}\right),c\leftarrow E_{sk_{lps}}\left(A_{ser}^{*},A_{ser}\right);$



store



      $\left(Z^{*},ku^{*},P^{*},A_{usr}^{*},t_{usr}^{*}\right)$


$\overset{\;(Z^{*},V^{*},P^{*},A_{sc}^{*},t_{sc}^{*})\;}{\underset{\;}{\leftarrow }}$


store



$\left(Z^{*},V^{*},P^{*},A_{sc}^{*},t_{sc}^{*}\right)$






$\overset{\;(t_{ser}^{*},c)\;}{\underset{\;}{\to }}$




compute



      $D_{sk_{lps}}\left(c\right)=\left(A_{ser}^{*},A_{ser}^{\prime }\right);$



if    $A_{ser}^{\prime }\neq A_{ser}$, stop;



store



      $\left(t_{ser}^{*},A_{ser}^{*}\right)$

#### 4.4.3. Rebuilding a New Smart Card

When the user’s smart card is unavailable, the user can use the laptop to interact with the server to rebuild it. The process of rebuilding a new smart card is as follows (see Game 7):

1. The user chooses a new identity of smart card $ID_{sc}^{*}$, a new random number $ku^{*}$, and a new first-degree polynomial $f^{*}\left(x\right)=r^{*}x+k$, where $r^{*}$ is a random number and two new symmetric keys are $sk_{lps}^{*}$ and $sk_{scs}^{*}$.
2. The user computes three true shares $y_{usr}^{*}=f^{*}\left(ID_{usr}\right)$, $y_{sc}^{*}=f^{*}\left(ID_{sc}^{*}\right)$, and $y_{ser}^{*}=f^{*}\left(ID_{ser}\right)$, three pseudoshares $A_{usr}^{*}=y_{usr}^{*}⊕Pwd⊕R1$, $A_{sc}^{*}=y_{sc}^{*}⊕Pwd⊕R1$, and $A_{ser}^{*}=y_{ser}^{*}⊕Pwd⊕R1$, the authentication message $V^{*}=h(ID_{sc}^{*}||Pwd||ID_{usr}||R1||ku^{*})$, and $t_{usr}^{*}=Mac_{R2}\left(A_{usr}^{*}\right)$, $t_{ser}^{*}=Mac_{R2}\left(A_{ser}^{*}\right)$, $t_{sc}^{*}=Mac_{R2}\left(A_{sc}^{*}\right)$.
3. The laptop uses $\left(A_{usr}^{*},t_{usr}^{*},ku^{*},sk_{lps}^{*}\right)$ to replace $\left(A_{usr},t_{usr},ku,sk_{lps}\right)$ and sends message $\left(t_{ser}^{*},c=E_{sk_{lps}}\left(A_{ser}^{*},A_{ser},sk_{lps}^{*},sk_{scs}^{*}\right),ID_{sc}^{*}\right)$ to the server.
4. After receiving the message $\left(t_{ser}^{*},c,ID_{sc}^{*}\right)$, the server calculates $D_{sk_{lps}}\left(c\right)=\left(A_{ser}^{*},A_{ser}^{\prime },sk_{lps}^{*},sk_{scs}^{*}\right)$ and checks if $A_{ser}^{\prime }$ is equal to $A_{ser}$. If $A_{ser}^{\prime }=A_{ser}$, the server replaces $\left(A_{ser},t_{ser},sk_{lps},sk_{scs},ID_{sc}\right)$ with $\left(A_{ser}^{*},t_{ser}^{*},sk_{lps}^{*},sk_{scs}^{*},ID_{sc}^{*}\right)$; otherwise, the server stops.
5. The laptop sends the message $\left(ID_{sc}^{*},A_{sc}^{*},t_{sc}^{*},V^{*},sk_{scs}^{*},Z,P\right)$ to the smart card.
6. The user stores $\left(ID_{sc}^{*},A_{sc}^{*},t_{sc}^{*},V^{*},sk_{scs}^{*},Z,P\right)$ in the new smart card.

**Game 7** Rebuilding a new smart cardNew smart card
Laptop (user)
Server

choose



      $ID_{sc}^{*},ku^{*},r^{*},sk_{lps}^{*},sk_{scs}^{*},$



      f* (*x*)=r*x + *k*;



compute



        $y_{usr}^{*}=f^{*}\left(ID_{usr}\right)$,



         $y_{sc}^{*}=f^{*}\left(ID_{sc}^{*}\right)$,



        $y_{ser}^{*}=f^{*}\left(ID_{ser}\right)$,



        $A_{usr}^{*}=y_{usr}^{*}⊕Pwd⊕R1$,



         $A_{sc}^{*}=y_{sc}^{*}⊕Pwd⊕R1$,



        $A_{ser}^{*}=y_{ser}^{*}⊕Pwd⊕R1$,



        $V^{*}=h(ID_{sc}^{*}||Pwd||ID_{usr}||R1||ku^{*})$,



      $t_{usr}^{*}\leftarrow Mac_{R2}\left(A_{usr}^{*}\right),$



     $t_{sc}^{*}\leftarrow Mac_{R2}\left(A_{sc}^{*}\right),$



     $t_{ser}^{*}\leftarrow Mac_{R2}\left(A_{ser}^{*}\right),$



     $c\leftarrow E_{sk_{lps}}\left(A_{ser}^{*},A_{ser},sk_{lps}^{*},sk_{scs}^{*}\right);$



store



      $\left(A_{usr}^{*},t_{usr}^{*},ku^{*},sk_{lps}^{*}\right)$




$\overset{\;(t_{ser}^{*},c,ID_{sc}^{*})\;}{\underset{\;}{\to }}$



compute



      $D_{sk_{lps}}\left(c\right)=\left(A_{ser}^{*},A_{ser}^{\prime },sk_{lps}^{*},sk_{scs}^{*}\right);$



if    $A_{ser}^{\prime }\neq A_{ser}$, stop;



store



      $\left(t_{ser}^{*},A_{ser}^{*},sk_{lps}^{*},sk_{scs}^{*},ID_{sc}^{*}\right)$
$\overset{\;(ID_{sc}^{*},A_{sc}^{*},t_{sc}^{*})\;}{\underset{\left(V^{*},sk_{scs}^{*},Z,P\right)}{\leftarrow }}$


store



$\left(ID_{sc}^{*},A_{sc}^{*},t_{sc}^{*},V^{*},sk_{scs}^{*},Z,P\right)$





#### 4.4.4. Rebuilding a New Laptop

When the user’s laptop is unavailable, the user can use the smart card to interact with the server to rebuild it. The process of rebuilding a new laptop is as follows (see Game 8):

1. The user chooses a new random number $ku^{*}$, a new first-degree polynomial $f^{*}\left(x\right)=r^{*}x+k$, where $r^{*}$ is a random number, and two new symmetric keys $sk_{lps}^{*}$ and $sk_{scs}^{*}$.
2. The user computes three true shares $y_{usr}^{*}=f^{*}\left(ID_{usr}\right)$, $y_{sc}^{*}=f^{*}\left(ID_{sc}\right)$, and $y_{ser}^{*}=f^{*}\left(ID_{ser}\right)$, three pseudoshares $A_{usr}^{*}=y_{usr}^{*}⊕Pwd⊕R1$, $A_{sc}^{*}=y_{sc}^{*}⊕Pwd⊕R1$, and $A_{ser}^{*}=y_{ser}^{*}⊕Pwd⊕R1$, the authentication message $V^{*}=h(ID_{sc}||Pwd||ID_{usr}||R1||ku^{*})$, and $t_{usr}^{*}=Mac_{R2}\left(A_{usr}^{*}\right)$, $t_{ser}^{*}=Mac_{R2}\left(A_{ser}^{*}\right)$, $t_{sc}^{*}=Mac_{R2}\left(A_{sc}^{*}\right)$.
3. The new laptop sends the message $\left(A_{sc}^{*},t_{sc}^{*},V^{*},sk_{scs}^{*}\right)$ to the smart card.
4. The smart card uses $\left(A_{sc}^{*},t_{sc}^{*},V^{*},sk_{scs}^{*}\right)$ to replace $\left(A_{sc},t_{sc},V,sk_{scs}\right)$ and sends the message $\left(Z,P,sk_{scs}^{*}\right)$ to the new laptop.
5. After receiving the message $\left(Z,P,sk_{scs}^{*}\right)$, the new laptop stores $\left(ID_{usr},A_{usr}^{*},t_{usr}^{*},ku^{*},sk_{lps}^{*},Z,P\right)$ and sends message $\left(t_{ser}^{*},c\leftarrow E_{sk_{scs}}\left(A_{ser}^{*},A_{ser},sk_{lps}^{*},sk_{scs}^{*}\right)\right)$ to the server.
6. After receiving the message $\left(t_{ser}^{*},c\right)$, the server computes $D_{sk_{scs}}\left(c\right)=\left(A_{ser}^{*},A_{ser}^{\prime },sk_{lps,}^{*}sk_{scs}^{*}\right)$ and checks whether $A_{ser}^{\prime }$ is equal to $A_{ser}$. If $A_{ser}^{\prime }=A_{ser}$, the server uses $\left(A_{ser}^{*},t_{ser}^{*},sk_{lps}^{*},sk_{scs}^{*}\right)$ to replace $\left(A_{ser},t_{ser},sk_{lps},sk_{scs}\right)$; otherwise, the server stops.

**Game 8** Rebuilding a new laptopSmart card
New laptop (user)
Server

choose



      $ku^{*},r^{*},sk_{lps}^{*},sk_{scs}^{*},$



     f*(*x*)=r*x + *k*;



compute



      $y_{usr}^{*}=f^{*}\left(ID_{usr}\right)$,



       $y_{sc}^{*}=f^{*}\left(ID_{sc}\right)$,



      $y_{ser}^{*}=f^{*}\left(ID_{ser}\right)$,



      $A_{usr}^{*}=y_{usr}^{*}⊕Pwd⊕R1$,



       $A_{sc}^{*}=y_{sc}^{*}⊕Pwd⊕R1$,



      $A_{ser}^{*}=y_{ser}^{*}⊕Pwd⊕R1$,



        $V^{*}=h(ID_{sc}||Pwd||ID_{usr}||R1||ku^{*})$,



      $t_{usr}^{*}\leftarrow Mac_{R2}\left(A_{usr}^{*}\right),$



      $t_{sc}^{*}\leftarrow Mac_{R2}\left(A_{sc}^{*}\right),$



      $t_{ser}^{*}\leftarrow Mac_{R2}\left(A_{ser}^{*}\right);$


$\overset{\;(A_{sc}^{*},t_{sc}^{*},V^{*},sk_{scs}^{*})\;}{\underset{\;}{\leftarrow }}$


store



$\left(A_{sc}^{*},t_{sc}^{*},V^{*},sk_{scs}^{*}\right)$




$\overset{\;\;\;\;\;(Z,P,sk_{scs}^{*})\;\;\;\;\;}{\underset{\;}{\to }}$




store



      $\left(ID_{usr},A_{usr}^{*},t_{usr}^{*},ku^{*},sk_{lps}^{*},Z,P\right)$



compute



      $c\leftarrow E_{sk_{scs}}\left(A_{ser}^{*},A_{ser},sk_{lps}^{*},sk_{scs}^{*}\right);$




$\overset{\;(t_{ser}^{*},c)\;}{\underset{\;}{\to }}$




compute



      $D_{sk_{scs}}\left(c\right)=\left(A_{ser}^{*},A_{ser}^{\prime },sk_{lps}^{*},sk_{scs}^{*}\right);$



if    $A_{ser}^{\prime }\neq A_{ser}$, stop;



store



      $\left(t_{ser}^{*},A_{ser}^{*},sk_{lps}^{*},sk_{scs}^{*}\right)$

## 5. Security Analysis and Performance Evaluation

In this section, we mainly analyze the security and performance of our scheme. Our scheme is secure against all secure goals claimed in Section 2.5.

### 5.1. Security Analysis

#### 5.1.1. Resist Replay Attacks

When the laptop or the smart card is unavailable, the user needs to complete mutual authentication with the server through another device. The attacker can participate in this interaction process and implement a replay attack. We claim that our scheme can resist replay attacks.

If the smart card is unavailable, the user needs to complete mutual authentication with the server to recover the private key. During the authentication phase, the laptop will send a message $\left(c,ID_{usr},T1\right)$ to the server, and the attacker may intercept the message and continuously send it to the server to carry out a replay attack. Our scheme resists the above replay attacks using timestamps. The specific process of resisting replay attacks is as follows:

(1) After receiving the message, the server first needs to determine that $T^{*}-T1\leq \Delta t$ is established.
(2) When the laptop sends message $\left(c,ID_{usr},T1\right)$ to the server for the first time, we have $T^{*}-T1\leq \Delta t$. If the attacker continues to send the message to the server, the server will terminate the authentication process when the time does not satisfy equation $T^{*}-T1\leq \Delta t$.

From the above analysis, it can be found that our scheme can resist replay attacks if the smart card is unavailable. The same analysis can also be applied to the situation where the attacker implements replay attacks when the laptop is unavailable.

#### 5.1.2. Resist Impersonation User Attacks

In this kind of attack, the attacker attempts to impersonate a legitimate user and interacts with the server. If the attacker wants to impersonate a legitimate user, the attacker must calculate the ciphertext c1 to pass the server’s verification. It should be noted that $c1\leftarrow E_{sk_{lps}}\left(Y\right)$, so the attacker must calculate a valid *Y* and a correct key $sk_{lps}$. We recall that $Y=Z⊕h(ID_{usr}||Pwd||R1),R\leftarrow Rep\left(Bio,P\right),R=\left(R1,R2\right)$. The security of the fuzzy extractor guarantees that R1 is almost uniformly distributed if the biometric information has a high enough entropy. Therefore, calculating *Y* requires the attacker knowing the correct password Pwd, obtaining the correct biometric information Bio, and obtaining the values of *Z* and *P* stored in the smart card or the laptop. According to the description of the attacker’s ability, it is impossible for the attacker to obtain both the password and biometric information while obtaining the smart card or the laptop device. Therefore, the attacker cannot impersonate a legitimate user.

#### 5.1.3. Resist Impersonation Server Attacks

In this kind of attack, the attacker attempts to impersonate a legitimate server and interacts with the user. When the adversary successfully impersonates the server, they may deceive users or steal sensitive information. For example, the adversary may also send incorrect information to the user, causing them to rebuild a wrong key.

We analyze the case where the adversary wants to impersonate a legitimate server by interacting with the user through the laptop. We review the process in Section 4.2.2. If the attacker wants to impersonate a legitimate server, the attacker needs to calculate a valid mac $t_{ser}$ and a correct ciphertext c2 to pass the user’s verification. We recall that $t_{ser}\leftarrow Mac_{R2}\left(A_{ser}\right),R\leftarrow Rep\left(Bio,P\right),R=\left(R1,R2\right)$ and $c2\leftarrow E_{sk_{lps}}\left(A_{ser}\right)$.

Since the biometric information Bio has enough entropy, through the security of the fuzzy extractor, R2 is almost uniformly distributed. Then, by the security of MAC, it is infeasible for the adversary to forge a valid tag $t_{ser}$ without biometric information Bio. Through the security of the encryption scheme, it is infeasible to forge a valid ciphertext c2 for message $A_{ser}$ without the encryption key $sk_{lps}$. Therefore, the adversary must obtain the biometric information Bio, the valid $A_{ser}$, the encryption key $sk_{lps}$, and the auxiliary string *P*. However, according to the description of the adversary’s abilities, it is impossible for the adversary to simultaneously corrupt the server, obtain biometric information, and obtain the laptop. A similar analysis can be used for the situation where the laptop is unavailable.

#### 5.1.4. Resist Malicious Servers

In this attack, the attacker can act as a malicious server sending incorrect information to the user, causing them to rebuild a wrong key. We consider the situation where the server sends incorrect messages to the laptop. A similar analysis can also be applied to the situation where the server sends incorrect messages to the smart card.

We recall that, in our scheme, the server sends the message $\left(c2,t_{ser}\right)$ to the laptop. After the laptop receives this message, the laptop calculates $A_{ser}^{\prime }\leftarrow Dec_{sk_{lps}}\left(c2\right)$ and $b\leftarrow Vrfy_{R2}\left(A_{ser}^{\prime },t_{ser}\right)$.

In the chapter on resisting impersonation server attacks, we have analyzed that the attacker cannot forge legitimate messages $\left(c2,t_{ser}\right)$ to pass the user’s verification. Furthermore, when the server sends mismatched c2 and $t_{ser}$ to the laptop, b=0. Therefore, the user can determine the correctness of the messages sent by the server by checking the value of *b*. Thus, our scheme can resist malicious servers.

#### 5.1.5. Resist Offline Guessing Attacks

There are two ways for the adversary to perform offline guessing password attacks. One way involves validating the value of *V*, and the other involves validating the value of *Z*. We will separately discuss how our scheme resists offline guessing attacks in these two scenarios.

(1) The adversary validates the value of V. In our scheme, V is stored in the smart card; thus, the adversary can only carry out offline guessing attacks in this way if he obtains the smart card. We recall that $V=h(ID_{sc}||Pwd||ID_{usr}||R1||ku)$ and ku is a random number stored in the laptop. It should be noted that the adversary cannot simultaneously obtain the smart card, the biometric information, and the laptop, which means the adversary cannot know both R1 and ku at the same time. Therefore, if the adversary wants to perform offline password-guessing attacks, he must guess the value of R1 or ku correctly. The security of the fuzzy extractor guarantees that R1 is almost uniformly distributed since the biometrc information has enough entropy, and through the randomness of ku, the adversary cannot guess *V* correctly in polynomial time.
(2) The adversary validates the value of Z. In our scheme, Z is stored in the smart card and the laptop, thus the adversary can only carry out offline guessing attacks in this way if he obtains the smart card or the laptop. We recall that $Z=H⊕Y=h(ID_{usr}||Pwd||R1)⊕Y$. We consider the worst-case scenario, where the adversary obtains the laptop, which means that the adversary obtains both *Z* and $ID_{usr}$. We note that the adversary cannot simultaneously obtain the smart card, obtain the laptop, and corrupt the server, which means that the adversary cannot know both R1 and *Y* at the same time. Therefore, if the adversary wants to perform offline password-guessing attacks, he must guess the value of R1 or *Y* correctly. The security of the fuzzy extractor and the randomness of *Y* guarantees that, the adversary cannot guess *Z* correctly in polynomial time.

### 5.2. Performance Evaluation

In this section, we will show the time consumption of each phase. The notations we used in this section are the same as those in Section 3. Since the time complexity of the exclusive-OR operation and concatenation operation is negligible, we do not take them into account. Table 3 shows the time cost for each phase of our scheme while Table 4 shows the comparison between our scheme and Yi et al. ’s scheme in terms of efficiency.

Compared with Yi et al.’s scheme, our scheme reduces two hash computations during the registration phase; reduces twenty hash computations, adds four symmetric encryption and decryption computations during the authentication phase; and reduces four hash computations during the updating phase. According to the literature [45], we have $1T_{S}=2.5T_{H}$. Therefore, our scheme consumes less time than Yi et al.’s scheme by $2T_{H}+20T_{H}+4T_{H}-4T_{S}=26T_{H}-4T_{S}=26T_{H}-4*2.5T_{H}=16T_{H}$.

In Table 5, we compare the computational cost and communication cost in the similar schemes. In the authentication protocol, the frequency of login and authentication is much higher than the frequency of user registration, and the update phase is only executed when the user has an update request, so we only consider the authentication phase. When evaluating the computational and communication costs of these schemes, we assume that the laptop identity $ID_{usr}$, smart card identity $ID_{sc}$, server identity $ID_{ser}$, password pwd, output of the hash function *H*, ciphertext of symmetric encryption algorithm, timestamp, and random number are 128 bits, and the length of the random number *R* generated by the fuzzy extractor is 256 bits. The computation time of the XOR operation can be ignored. According to [4,45,46,47], we have $T_{H}\approx 9.18$ ms, $T_{S}=2.5T_{H}\approx 22.95$ ms, $T_{V}\approx 18.85$ ms, and $T_{R}\approx 63.08$ ms.

From the comparison results, it can be seen that the computational complexity of Liu et al.’s and Hu et al.’s schemes is significantly lower than the latter two schemes. However, as mentioned earlier (in the introduction), their protocol cannot achieve the security they claim. Their protocol’s small computational costs stems from a significant sacrifice in security. Our scheme achieves the same level of security as Yi et al.’s scheme, while also having higher efficiency. In addition, our scheme also reduces one round of communication in the authentication phase, which greatly reduces our communication costs. Among the four schemes, our scheme has the lowest communication cost.

## 6. Conclusions

This paper gave a systematic analysis of Yi et al.’s scheme and found that its efficiency is relatively low, because users need to establish a session key with the server using a laptop or a smart card before obtaining information stored on the server. This process involves a large amount of hash operations, and the above steps are repeated every time the update operation is performed. Therefore, we proposed a data backup scheme with better performance. Our scheme involves negotiating session keys in advance during the registration phase. In this way, we can reduce ten hash computations and one round of communication in the authentication phase. The experimental results show that our scheme has better execution efficiency and lower communication costs.

Meanwhile, this paper also carried out the security analysis of the scheme and ensured that the scheme has the same security as the scheme of Yi et al. [7].

## Figures and Tables

**Table 1:** The main abbreviation used in the paper.

Abbreviation	Meaning
$ID_{usr}$	Identity of the user
$ID_{sc}$	Identity of the smart card
$ID_{ser}$	Identity of the server
Pwd	Password of the user
Bio	Biometrics of the user
sk	Session key
ku	A random number selected by the user
X1,X2	Random number generated by the server
Ti	Time stamp
Δt	Time interval
⊕	Exclusive-or operation
${\#}^{*}$	A new message #
‖	Concatenation operator
h(.)	Collision-resistant hash function
A	Adversary
E/D	Symmetric encryption / decryption algorithm
R	The set of natural real numbers

**Table 2:** Computational cost of Yi et al.’s scheme.

Phase	Entity	$T_{H}$	$T_{S}$	$T_{P}$	$T_{M}$	$T_{V}$	$T_{G}$	$T_{R}$	$T_{\mathrm{SS}}$
Registration	Laptop	3	0	0	3	0	1	0	1
	Smart card	0	0	0	0	0	0	0	0
	Server	2	0	0	0	0	0	0	0
Authentication (Case 1)	Laptop	0	0	0	0	1	0	1	0
	Smart card	1	0	0	0	1	0	0	0
	Server	0	0	0	0	0	0	0	0
Authentication (Case 2)	Laptop	6	1	0	0	1	0	1	0
	Smart card	0	0	0	0	0	0	0	0
	Server	6	1	0	0	0	0	0	0
Authentication (Case 3)	Laptop	0	0	0	0	0	0	0	0
	Smart card	6	1	0	0	1	0	1	0
	Server	6	1	0	0	0	0	0	0
Rebuilding smart card	Laptop	1	1	0	3	0	0	0	1
	Smart card	0	0	0	0	0	0	0	0
	Server	0	1	0	0	0	0	0	0
Rebuilding laptop	Laptop	1	1	0	3	0	0	0	1
	Smart card	0	0	0	0	0	0	0	0
	Server	0	1	0	0	0	0	0	0
Updating biometrics	Laptop	5	1	0	3	0	1	1	0
	Smart card	1	0	0	0	0	0	0	0
	Server	0	1	0	0	0	0	0	0
Updating password	Laptop	5	1	0	3	0	0	1	0
	Smart card	1	0	0	0	0	0	0	0
	Server	0	1	0	0	0	0	0	0
Reconstruction	Laptop	0	0	1	0	0	0	0	0
	Smart card	0	0	0	0	0	0	0	0
	Server	0	0	0	0	0	0	0	0

**Table 3:** Computational cost of our scheme.

Phase	Entity	$T_{H}$	$T_{S}$	$T_{P}$	$T_{M}$	$T_{V}$	$T_{G}$	$T_{R}$	$T_{\mathrm{SS}}$
Registration	Laptop	2	0	0	3	0	1	0	1
	Smart card	0	0	0	0	0	0	0	0
	Server	1	0	0	0	0	0	0	0
Authentication (Case 1)	Laptop	0	0	0	0	1	0	1	0
	Smart card	1	0	0	0	1	0	0	0
	Server	0	0	0	0	0	0	0	0
Authentication (Case 2)	Laptop	1	2	0	0	1	0	1	0
	Smart card	0	0	0	0	0	0	0	0
	Server	1	2	0	0	0	0	0	0
Authentication (Case 3)	Laptop	0	0	0	0	0	0	0	0
	Smart card	1	2	0	0	1	0	1	0
	Server	1	2	0	0	0	0	0	0
Rebuilding smart card	Laptop	1	1	0	3	0	0	0	1
	Smart card	0	0	0	0	0	0	0	0
	Server	0	1	0	0	0	0	0	0
Rebuilding laptop	Laptop	1	1	0	3	0	0	0	1
	Smart card	0	0	0	0	0	0	0	0
	Server	0	1	0	0	0	0	0	0
Updating biometrics	Laptop	3	1	0	3	0	1	1	0
	Smart card	1	0	0	0	0	0	0	0
	Server	0	1	0	0	0	0	0	0
Updating password	Laptop	3	1	0	3	0	0	1	0
	Smart card	1	0	0	0	0	0	0	0
	Server	0	1	0	0	0	0	0	0
Reconstruction	Laptop	0	0	1	0	0	0	0	0
	Smart card	0	0	0	0	0	0	0	0
	Server	0	0	0	0	0	0	0	0

**Table 4:** Performance comparison between the proposed scheme and Yi et al.’s scheme.

Scheme	Compution Cost								
	Registration Phase			Authentication Phase			Updating Phase		
	Laptop	Smart Card	Server	Laptop	Smart Card	Server	Laptop	Smart Card	Server
Yi et al.’s scheme [7]	$3T_{H}+3T_{M}+1T_{G}+1T_{SS}$	0	$2T_{H}$	$6T_{H}+1T_{S}+2T_{V}+2T_{R}$	$7T_{H}+1T_{S}+2T_{V}+1T_{R}$	$12T_{H}+2T_{S}$	$12T_{H}+4T_{S}+12T_{M}+1T_{G}+2T_{R}+2T_{SS}$	$2T_{H}$	$4T_{S}$
Our scheme	$2T_{H}+3T_{M}+1T_{G}+1T_{SS}$	0	$1T_{H}$	$1T_{H}+2T_{S}+2T_{V}+2T_{R}$	$2T_{H}+2T_{S}+2T_{V}+1T_{R}$	$2T_{H}+4T_{S}$	$8T_{H}+4T_{S}+12T_{M}+1T_{G}+2T_{R}+2T_{SS}$	$2T_{H}$	$4T_{S}$
Time cost reduction		$2T_{H}$			$20T_{H}-4T_{S}=10T_{H}$			$4T_{H}$	

**Table 5:** Performance comparison between the proposed scheme and similar schemes.

Scheme	Authentication Phase	Entity			Execution Time	Communication Cost
		Laptop	Smart Card	Server	(ms)	(bits)
Liu et al.’s [5]	Case1	-	$T_{H}$	-	9.18	128
	Case2	$4T_{H}+T_{S}$	-	$5T_{H}+T_{S}$	128.52	896
	Case3	$4T_{H}+T_{S}$	$T_{H}$	$5T_{H}+T_{S}$	128.52	1024
Hu et al.’s [6]	Case1	$2T_{H}$	-	-	18.36	128
	Case2	$6T_{H}+T_{S}$	-	$4T_{H}+T_{S}$	137.70	1024
	Case3	$6T_{H}+T_{S}$	$2T_{H}$	$4T_{H}+T_{S}$	156.06	1024
Yi et al.’s [7]	Case1	$T_{V}+T_{R}$	$T_{H}+T_{V}$	-	109.96	768
	Case2	$6T_{H}+T_{S}+T_{V}+T_{R}$	-	$6T_{H}+T_{S}$	237.99	1152
	Case3	-	$6T_{H}+T_{S}+T_{V}+T_{R}$	$6T_{H}+T_{S}$	237.99	1152
Ours	Case1	$T_{V}+T_{R}$	$T_{H}+T_{V}$	-	109.96	768
	Case2	$1T_{H}+2T_{S}+T_{V}+T_{R}$	-	$1T_{H}+2T_{S}$	192.09	640
	Case3	-	$1T_{H}+2T_{S}+T_{V}+T_{R}$	$1T_{H}+2T_{S}$	192.09	640

## Data Availability

The original contributions presented in the study are included in the article, further inquiries can be directed to the corresponding author.

## Appendix A

In this section, we review Yi et al.’s scheme [7]. Their scheme consists of four phases: the registration phase, authentication phase, key reconstruction phase, and updating phase.

### Appendix A.1. Registration Phase

In this phase, the user inserts their smart card into the card reader and enters their identity, password, and biometrics. After initial calculations, the corresponding information is distributed to laptops, smart cards, and servers for storage:

1. The user chooses a robust fuzzy extractor (Gen,Rep), a message authentication code (Gen,Mac,Vrfy), and a hash function *h*. The user chooses $ID_{usr},ID_{sc},ID_{ser},Pwd,Bio$, a random number ku, and a first-degree polynomial f(x)=rx+k, where *k* is the key to encrypt sensitive information, and *r* is a random number.
2. The user computes (R,P)←Gen(Bio), and divides *R* into two parts R1 and R2, which are, respectively, applied to the three pseudoshares and the message authentication code. The user computes three true shares $y_{usr}=f\left(ID_{usr}\right),y_{sc}=f\left(ID_{sc}\right),$ and $y_{ser}=f\left(ID_{ser}\right)$, the three pseudoshares $A_{usr}=y_{usr}⊕Pwd⊕R1$, $A_{sc}=y_{sc}⊕Pwd⊕R1$, and $A_{ser}=y_{ser}⊕Pwd⊕R1$, the authentication message $V=h(ID_{sc}||Pwd||ID_{usr}||R1||ku)$, $C1=h(ID_{usr}||Pwd||R1)$, $C2=h(ID_{ser}||Pwd||R1)$, and three tags $t_{usr}\leftarrow Mac_{R2}\left(A_{usr}\right)$, $t_{sc}\leftarrow Mac_{R2}\left(A_{sc}\right)$, and $t_{ser}\leftarrow Mac_{R2}\left(A_{ser}\right)$.
3. The user sends a message $\left(A_{ser},ID_{ser},C1,C2,t_{ser}\right)$ to the server.
4. After the server receives the message $\left(A_{ser},ID_{ser},C1,C2,t_{ser}\right)$, the server chooses two random numbers X1 and X2 and generates $Y1=h(ID_{ser}||X1),Y2=h(ID_{ser}||X2),Z1=Y1⊕C1$ and Z2=Y2⊕C2. Finally, the server stores values $\left(X1,X2,A_{ser},ID_{ser},t_{ser}\right)$.
5. The server sends the messages (Z1,Z2) to the user.
6. The user stores values $\left(A_{usr},ID_{usr},t_{usr},P,Z1,Z2,ku\right)$ in the laptop.
7. The user then sends $\left(A_{sc},ID_{sc},t_{sc},V,Z1,Z2,P\right)$ to the smart card.
8. The smart card stores $\left(A_{sc},ID_{sc},t_{sc},V,Z1,Z2,P\right)$.

### Appendix A.2. Authentication Phase

The authentication phase contains three situations: possession of the laptop and the smart card, losing the smart card, and losing the laptop. After performing the authentication phase, the user obtains the information required to recover the key.

#### Appendix A.2.1. Possession of the Laptop and the Smart Card

The users complete the authentication phase through the interaction between the laptop and the smart card. The specific process is as follows:

1. The user inserts the smart card into the card reader, and the card reader reads the information $\left(A_{sc},ID_{sc},t_{sc},V,Z1,Z2,P\right)$ in the smart card.
2. The user inputs $Bio^{*}$, and computes $Rep\left(P,Bio^{*}\right)=R^{*}$, then divides $R^{*}$ into two parts $R1^{*}$ and $R2^{*}$. The smart card verifies whether $t_{sc}$ is a valid tag for message $A_{sc}$, $b\leftarrow Vrfy_{R2^{*}}\left(t_{sc},A_{sc}\right)$. If b=0, the smart card stops; otherwise, the laptop verifies whether $t_{usr}$ is a valid tag for message $A_{usr}$, $b\leftarrow Vrfy_{R2^{*}}\left(t_{usr},A_{usr}\right)$. If b=0, the laptop stops; otherwise, it continues.
3. The user inputs their $ID_{usr}$ and Pwd to the smart card, and the laptop sends ku to the smart card.
4. The smart card computes $V^{*}=h(ID_{sc}||Pwd||ID_{usr}||R1^{*}\left||ku\right)$, then checks if $V^{*}$ is equal to *V*.
5. If $V^{*}\neq V$, the procedure aborts; otherwise, the smart card sends $\left(R1^{*},A_{sc}\right)$ to the laptop.
6. After receiving $\left(A_{sc},R1^{*}\right)$, the user computes $y_{sc}^{*}=A_{sc}⊕Pwd⊕R1^{*},y_{usr}^{*}=A_{usr}⊕Pwd⊕R1^{*}$.

#### Appendix A.2.2. Losing the Smart Card

When the user loses the smart card, he needs to complete the authentication phase through the interaction between the laptop and the server. The specific process is as follows:

Mutual authentication between the user and the server:

1. The user inputs $Bio^{*}$, computes $Rep\left(P,Bio^{*}\right)=R^{*}$ and divides $R^{*}$ into two parts $R1^{*}$ and $R2^{*}$. Then, the user inputs their $ID_{usr},Pwd$. The laptop computes $Y1=Z1⊕h(ID_{usr}||Pwd||R1^{*}),Y2=Z2⊕h(ID_{ser}||Pwd||R1^{*}),m4=Y1⊕Ru$ and VC1=h(Y2||Ru||T1), where Ru is a random number chosen by the laptop, and T1 is the current timestamp.
2. The laptop sends (VC1,m4,T1) to the server.
3. After receiving the request message (VC1,m4,T1), the server checks whether the current timestamp $T^{*}-T1\leq \Delta t$ or not. If $T^{*}-T1>\Delta t$, the server stops; otherwise, the server computes $Y1=h(ID_{ser}||X1),Y2=h(ID_{ser}||X2),m5=m4⊕Y1$ and checks whether h(Y2||m5||T1) is equal to VC1 or not. If they are not equal, the server stops; otherwise, the server authenticates the user. Then, the server chooses a random number Rs, and computes $m6=Y1⊕Y2⊕Rs,sk_{s}=h(VC1||m5||Rs),VC2=h(sk_{s}\left||Y2||Rs||T2\right)$, where T2 is the current timestamp.
4. The server sends message (VC2,m6,T2) to the laptop.
5. After receiving the response message (VC2,m6,T2), the laptop checks whether the current timestamp $T^{*}-T2\leq \Delta t$ or not. If $T^{*}-T2>\Delta t$, the laptop stops; otherwise, the laptop computes $Rs=m6⊕Y1⊕Y2,sk_{u}=h\left(VC1||Ru||Rs\right)$ and checks whether $h(sk_{u}||Y2||Rs||T2)$ is equal to VC2. If they are equal, the user authenticates the server; otherwise, the laptop stops.

Establishing a session key between the user and the server:

1. The laptop computes $CM1=h(sk_{u}||Y1||Y2||VC1||VC2||Ru||m6⊕Y1⊕Y2)$ and sends CM1 to the server.
2. After receiving the message CM1, the server checks whether $h(sk_{s}||Y1||Y2||VC1||VC2||m4⊕Y1||Rs)$ is equal to CM1 or not. If they are equal, it sets the session key $sk=sk_{s}=sk_{u}$. The server computes $c\leftarrow E_{sk}\left(A_{ser}\right)$ and sends $\left(c,t_{ser}\right)$ to the laptop.
3. After receiving the message $\left(c,t_{ser}\right)$, the user computes $A_{ser}^{\prime }\leftarrow Dec_{sk}\left(c\right)$. The user computes $b\leftarrow Vrfy_{R2^{*}}\left(A_{ser}^{\prime },t_{ser}\right)$. If b=0, the user stops; otherwise, the user computes $y_{ser}=A_{ser}^{\prime }⊕R1^{*}⊕Pwd$.

#### Appendix A.2.3. Losing the Laptop

The process is similar to the case of losing the smart card.

### Appendix A.3. Key Reconstruction Phase

When the user has two of three tuples:$\left(y_{sc},ID_{sc}\right),\left(y_{ser},ID_{ser}\right)$, and $\left(y_{usr},ID_{usr}\right)$, he can easily reconstruct the key *k* by using the Lagrangian interpolation polynomial.

### Appendix A.4. Updating Phase

During the updating phase, the user can rebuild their smart cards, laptops, or update passwords and biometrics.

#### Appendix A.4.1. Updating Password

The specific process is as follows:

1. The user inserts the smart card into the card reader, and the card reader reads the information in the smart card.
2. The user inputs biometrics Bio, the old password Pwd, and obtains the old random number ku,$ID_{usr}$ in the laptop and the $ID_{sc}$ in the smart card.
3. The user computes (R,P)←Gen(Bio), and divides *R* into two parts R1 and R2. The user checks if $h(ID_{sc}||Pwd||ID_{usr}||R1||ku)$ is equal to *V*. If they are equal, the user continues; otherwise, the user stops.
4. The user chooses a new random number $ku^{\prime }$ and a new password $Pwd^{*}$ and computes following values: $V^{*}=h(ID_{sc}||Pwd^{*}||ID_{usr}||R1||ku^{\prime })$
  $C1^{*}=h(ID_{usr}||Pwd^{*}||R1),C2^{*}=h(ID_{ser}||Pwd^{*}\left||R1\right)$
  $C1=h(ID_{usr}||Pwd||R1),C2=h(ID_{ser}\left||Pwd||R1\right)$
  $Z1^{*}=Z1⊕C1⊕C1^{*},Z2^{*}=Z2⊕C2⊕C2^{*}$
  Then the user stores $\left(Z1^{*},Z2^{*},ku^{\prime }\right)$ in the laptop to replace (Z1,Z2,ku) and stores $\left(Z1^{*},Z2^{*},V^{*}\right)$ in the smart card to replace (Z1,Z2,V).

#### Appendix A.4.2. Updating Biometrics

The process is similar to the case of updating the password.

#### Appendix A.4.3. Rebuilding a New Smart Card

The user first needs to establish a session key sk with the server, which is consistent with the loss of the smart card during the authentication phase. After the user establishes the session key, the specific process is as follows:

1. The user chooses a new identity of the smart card $ID_{sc}^{*}$, a new random number $ku^{*}$, and a new first-degree polynomial $f^{*}\left(x\right)=r^{*}x+k$, where $r^{*}$ is a random number.
2. The user computes three true shares $y_{usr}^{*}=f^{*}\left(ID_{usr}\right),y_{sc}^{*}=f^{*}\left(ID_{sc}^{*}\right),$ and $y_{ser}^{*}=f^{*}\left(ID_{ser}\right)$, three pseudoshares $A_{usr}^{*}=y_{usr}^{*}⊕Pwd⊕R1,A_{sc}^{*}=y_{sc}^{*}⊕Pwd⊕R1,$ and $A_{ser}^{*}=y_{ser}^{*}⊕Pwd⊕R1$, the authentication message $V^{*}=h(ID_{sc}^{*}||Pwd||ID_{usr}||R1||ku^{*})$, and $t_{usr}^{*}=Mac_{R2}\left(A_{usr}^{*}\right),t_{ser}^{*}=Mac_{R2}\left(A_{ser}^{*}\right),$ and $t_{sc}^{*}=Mac_{R2}\left(A_{sc}^{*}\right)$.
3. The laptop uses $\left(A_{usr}^{*},t_{usr}^{*},ku^{*}\right)$ to replace $\left(A_{usr},t_{usr},ku\right)$ and sends message $\left(c=E_{sk}\left(A_{ser}^{*},A_{ser}\right),t_{ser}^{*}\right)$ to the server.
4. After receiving the message $\left(c,t_{ser}^{*}\right)$, the server computes $D_{sk}\left(c\right)=\left(A_{ser}^{*},A_{ser}^{\prime }\right)$ and checks if $A_{ser}^{\prime }$ is equal to $A_{ser}$. If $A_{ser}^{\prime }=A_{ser}$, the server uses $\left(A_{ser}^{*},t_{ser}^{*}\right)$ to replace $\left(A_{ser},t_{ser}\right)$. Otherwise, the server stops.
5. The user stores $\left(ID_{sc}^{*},A_{sc}^{*},t_{sc}^{*},V^{*},Z1,Z2\right)$ in the smart card.

#### Appendix A.4.4. Rebuilding a New Laptop

The process is similar to the case of rebuilding a smart card.
